# Glucose and Lipid Profiles of Overweight and Obese Children in Riyadh, Saudi Arabia

**DOI:** 10.7759/cureus.38902

**Published:** 2023-05-11

**Authors:** Mohammed A AlAteeq, AbdulAziz AlHusaini, AbdulAziz AlMalahi, Faisal AlOtay, Azzam AlTurki, AbdulAziz Alghafis

**Affiliations:** 1 Family Medicine, King Abdullah International Medical Research Center, King Saud Bin Abdulaziz University for Health Sciences, National Guard Health Affairs, Riyadh, SAU; 2 Medicine, King Saud Bin Abdulaziz University for Health Sciences, National Guard Health Affairs, Riyadh, SAU

**Keywords:** dyslipidemia, atherosclerotic disease, prevention, pediatrics, metabolic diseases

## Abstract

Background: Obesity is a prevalent issue worldwide, affecting both children and adults and posing significant health risks. Obesity and overweight are known to be associated with metabolic abnormalities among children and adolescents. This study aims to determine the metabolic profiles, identifying any abnormalities and related factors among overweight and obese children in Saudi Arabia (SA).

Methods: This study conducted a cross-sectional, descriptive, and analytical analysis on 382 overweight and obese children between the ages of seven and 14 years. The subjects were visitors to pediatric endocrinology clinics and primary healthcare clinics in King Abdulaziz Medical City (KAMC) in Riyadh, Saudi Arabia. Data from the electronic medical records between 2018 and 2020 were examined, focusing on total cholesterol (TC), low-density lipoprotein cholesterol (LDL-C), high-density lipoprotein cholesterol (HDL-C), triglycerides (TG), and fasting blood sugar (FBS).

Results: Among the study sample, 8% were found to have high TC, 19% had high LDL-C, 27% had low HDL-C, 12% had high TG, and 8% had high FBS. Overweight children had higher HDL levels, while obese children had higher TG levels. There was no significant difference between males and females or between different age groups in metabolic profiles.

Conclusion: This study uncovered a low prevalence of abnormal lipid and FBS profiles among overweight and obese children and adolescents. Detecting and managing the early onset of dyslipidemia and hyperglycemia can prevent long-term consequences and safeguard children from the risk of future cardiovascular injuries and deaths.

## Introduction

Obesity, characterized by excessive fat accumulation, is a significant worldwide health issue affecting children and adults. It poses various health hazards, causing impairments in physical and psychological activities [1]. Additionally, obesity significantly contributes to chronic noncommunicable diseases such as type 2 diabetes mellitus, dyslipidemia, and hypertension and a 2.57-fold increase in mortality risk [2].

Obesity and overweight are increasingly prevalent among children, especially in economically developed countries and urbanized populations, making them significant global health concerns [3]. Newly published data showed an increase in the prevalence of obesity and overweight among children and adolescents, especially in developing countries [4].

The incidence of overweight and obesity among children in Saudi Arabia (SA) has also increased in recent years. Several studies have documented regional differences in the prevalence of obesity and overweight among Saudi children, but overall, the incidence is rising [5-11]. In 2015, a study of 7,930 children aged 6-16 years in Riyadh, SA, found an overall prevalence of 13.4% for overweight and 18.2% for obesity [12]. According to other epidemiological studies, the percentage of overweight and obese children aged 6-12 years is now over 23% and 9.3%, respectively, while for preschool children, the rates are approximately 15% for overweight and 6% for obesity [5]. Another cross-sectional study conducted in Al-Qassim Province compared prevalence data from 2012 to older data from 1994 to 1998 and revealed a significant increase in the number of overweight children from 10.1% to 16.9% [6].

Obesity and overweight are commonly associated with metabolic abnormalities among children and adolescents [13-16]. A systematic review investigating the relationship between abdominal obesity and heart-related risk factors in children and adolescents found that central body fat deposition raised the risk of cardiometabolic risk factors [17]. In the United States, a report evaluating cardiometabolic risk factors in extremely obese children and adolescents from 1999 to 2012 showed that the prevalence of high total cholesterol (TC), low high-density lipoprotein (HDL), high triglycerides (TG), high low-density lipoprotein (LDL), and high fasting glucose was 16.5%, 40.0%, 30.0%, 13.0%, and 26.8%, respectively [13]. A related study of 263 Lebanese adolescents found that metabolic syndrome (MS) was present in 21.2% of obese and 3.8% of overweight participants. High waist circumference (96.2%), low HDL (96.2%), and hypertriglyceridemia were the primary metabolic disorders among subjects with MS (73.1%) [14].

In Saudi Arabia, a local study demonstrated that a high body mass index (BMI) was linked to increased LDL levels and decreased HDL levels in children aged two to 18 years old [18]. Among children in Riyadh recently diagnosed with diabetes, a study showed that 64% were overweight or obese, and 34% exhibited signs of insulin resistance [19]. A similar study conducted on 200 children from Jeddah, western Saudi Arabia, reported comparable findings [20].

Limited research has been conducted in Saudi Arabia on metabolic changes in overweight and obese children. This study aims to evaluate the extent of metabolic abnormalities and identify related factors among overweight and obese Saudi children.

## Materials and methods

This study was a cross-sectional, descriptive, and analytical investigation that examined 382 Saudi children of both genders, aged 7-14 years, with BMI above the 85th percentile but less than the 95th percentile for overweight and 95th percentile and above for obesity.

The study population comprised visitors to pediatric endocrinology clinics and three main family medicine and primary healthcare clinics at King Abdulaziz Medical City (KAMC) in Riyadh, Saudi Arabia.

To calculate the sample size, Raosoft online sample size calculator (Raosoft, Inc., Seattle, WA) was used, considering a study population of approximately 50,000, with an expected abnormality rate of 50%, a 5% margin of error, and a 95% confidence interval. The estimated sample size was 382. The non-probability convenience sampling technique was utilized to select the study sample from a list of children registered in the system as having obesity or overweight between 2018 and 2020.

The exclusion criteria consisted of children with type 1 or type 2 diabetes mellitus, familial hyperlipidemia, chronic kidney disease, endocrine disorders, eating disorders, and genetic syndromes; patients taking dyslipidemia or psychiatric medications; and those receiving systemic corticosteroids.

The Institutional Review Board (IRB) of King Abdullah International Medical Research Center, Riyadh, issued approval number SP21R/246/05 dated 28 June 2021.

Upon study approval, the available data of study subjects between 2018 and 2020 were extracted from the electronic medical records system (BESTCare, ezCaretech, Seoul, South Korea). A data collection sheet was designed to fulfill the study objectives and included three sections: section 1 for demographic data (age, gender, chronic medical conditions, and current medications), section 2 for anthropometric indices (weight, height, and BMI), and section 3 for the latest laboratory results for fasting blood sugar (FBS) and lipid profiles (total cholesterol {TC}, low-density lipoprotein cholesterol {LDL-C}, high-density lipoprotein cholesterol {HDL-C}, and triglyceride {TG}).

An abnormal FBS was defined as levels above 5.6 mmol/L in accordance with the American Diabetes Association guidelines [21]. Lipid profile abnormalities were based on the values reported by the National Cholesterol Education Program Expert Panel on Cholesterol Levels in Children [22]. High TC levels were considered above 5.2 mmol/L, consistent with the >95th percentile of serum cholesterol in Saudi children, as reported by Rafii et al. [23]. LDL-C levels were considered high if they exceeded 3.4 mmol/L, while low HDL-C was defined as below 1 mmol/L. TG levels were deemed high if they exceeded 1.1 mmol/L and 1.5 mmol/L for the age groups 7-9 years and 10-14 years, respectively.

The data were entered and analyzed using the Statistical Package for Social Sciences (SPSS) software (IBM SPSS Statistics, Armonk, NY). Descriptive statistics were performed for categorical variables, and the mean and standard deviation were used for continuous variables. Analytical statistics were conducted using chi-square tests to evaluate the differences between categorical variables and independent Student's t-tests to compare means, with a statistical significance level set at 0.05 or less.

## Results

The study analyzed the profiles of 382 children with a mean age of 12.4 (±1.6) years. Most of the study subjects were above 13 years of age (34.3%), and 50.5% were females. Most subjects were obese (84.6%), while 15.4% were overweight. The minimum, maximum, and mean BMI were 25.05, 53.81, and 32.3 (±5.3), respectively. Table 1 summarizes the patient characteristics.

**Table 1:** Subject's characteristics (n=382) BMI: body mass index

Subject's demographics		N	%
Age (years)	7-10	47	12.3%
	11	67	17.5%
	12	66	17.3%
	13	71	18.6%
	14	131	34.3%
Gender	Male	189	49.5%
	Female	193	50.5%
BMI	Overweight	59	15.4%
	Obese	323	84.6%

Table 2 presents the metabolic profiles of the study sample, with 8% having high TC, 19% having high LDL, 27% having low HDL, 12% having high TG, and 8% having high FBS.

**Table 2:** Abnormal metabolic profiles BMI, body mass index; TC, total cholesterol; LDL, low-density lipoprotein; HDL, high-density lipoprotein; TG, triglycerides; FBS, fasting blood sugar

Metabolic profiles	BMI groups		Total	%
	Overweight	Obese		
High TC	12	19	31	8%
High LDL	28	44	72	19%
Low HDL	29	73	102	27%
High TG (general)	15	30	45	12%
High TG (ages 0-9)	4	2	6	1.5%
High TG (ages 9-14)	11	28	39	10%
High FBS	8	24	32	8%

The minimum, maximum, and mean values for metabolic profiles are presented in Table 3.

**Table 3:** Minimum, maximum, mean, and SD of metabolic profiles (n=382) TC, total cholesterol; LDL, low-density lipoprotein; HDL, high-density lipoprotein; TG, triglycerides; FBS, fasting blood sugar; SD, standard deviation

Metabolic profiles	Minimum	Maximum	Mean	SD
TC (mmol/L)	1.64	7.49	4.2	0.8
LDL (mmol/L)	0.3	6.4	2.8	0.8
HDL (mmol/L)	0.13	4.08	1.2	0.3
TG (mmol/L)	0.15	3.41	1.0	0.4
FBS (mmol/L)	3.4	7.5	5.0	0.5

Overweight children had higher HDL levels, while obese children had higher TG levels. There was no significant difference between males and females or among different age groups in metabolic profiles (Table 4).

**Table 4:** Demographics versus metabolic profiles (n=382) BMI, body mass index; TC, total cholesterol; LDL, low-density lipoprotein; HDL, high-density lipoprotein; TG, triglycerides; FBS, fasting blood sugar; SD, standard deviation

Subject's demographics		TC		LDL		HDL		TG		FBS	
		Mean	SD	Mean	SD	Mean	SD	Mean	SD	Mean	SD
Age	7-10	4.27	0.72	2.9	0.77	1.14	0.27	1.05	0.48	5.11	0.37
	11	4.2	0.9	2.73	0.9	1.17	0.25	1.03	0.51	5.04	0.43
	12	4.34	0.77	3.05	0.8	1.17	0.25	0.99	0.39	5.04	0.53
	13	4.1	0.68	2.71	0.7	1.21	0.44	1	0.35	5.05	0.53
	14	4.14	0.72	2.79	0.72	1.11	0.19	1.01	0.4	5.01	0.49
	P-value	0.326		0.063		0.176		0.935		0.797	
Gender	Male	4.26	0.7	2.84	0.69	1.16	0.24	1.05	0.44	5.04	0.44
	Female	4.13	0.8	2.81	0.85	1.15	0.32	0.97	0.4	5.04	0.51
	P-value	0.107		0.715		0.829		0.056		0.995	
BMI groups	Overweight	4.22	0.88	2.8	0.9	1.23	0.21	0.87	0.31	5.01	0.53
	Obese	4.19	0.73	2.83	0.75	1.14	0.29	1.04	0.44	5.04	0.47
	P-value	0.752		0.82		0.031		0.005		0.658	

## Discussion

The study provides a recent and detailed description of the glucose and lipid profiles among overweight and obese Saudi children aged 7-14 years.

The majority of the study sample had obesity (84.6%), reflecting a growing health issue that requires serious consideration. According to Al-Hussaini et al.'s study published in 2019, the latest reported prevalence of obesity among Saudi children was 18.2% (18% for females and 18.4% for males; P=0.73) [12]. As shown by Al Shehri et al. in their review article, Saudi children have a high prevalence of obesity and overweight, with a rising trend and regional variation across Saudi Arabia [5]. Al-Muhaimeed et al. also reported a similar increasing trend over the last 15 years [6]. This trend is partially attributed to changing lifestyles, with younger generations consuming more fast food [11]. Alturki et al. demonstrated that the frequency of fast-food intake was higher among obese children than their normal-weight peers [7].

The study results showed a low rate of lipid and glucose abnormality among the study sample. For comparison, in a study of severely obese American children and adolescents, the prevalence of abnormally elevated LDL-C was 7.45%, which is much less than that reported in the current study. On the other hand, the prevalence of high FBS in the current study was much lower than that reported in the former study (13.65%), while the prevalence of abnormal TC and TG and mean lipid profiles and FBS was almost the same [13]. Similarly, in the Bogalusa Heart Study of overweight and obese American children aged 4-18 years, the means for lipid profiles were comparable to the current study findings, while the mean FBS was lower [15]. In a regional study conducted in Turkey, a similar prevalence of high LDL was found among obese children, while the rates of high TC and TG were higher [24]. In Portugal, a similar study conducted on obese children found a mean TC of 4.11 mmol/L, LDL-C of 2.63 mmol/L, TG of 0.86 mmol/L, and FBS of 5 mmol/L [16]. These results are consistent with our findings, despite the difference in mean BMI, which was higher in the current study (29.8 versus 32.3). Another similar study conducted on obese children in Germany reported much higher rates of lipid profile abnormalities, although the mean BMI was less than that reported in the current study. In the aforementioned study, high TC, LDL-C, and TG levels were found in 38.5%, 27.2%, and 33.9%, respectively [25]. This difference may be related to the difference in ethnicity between the two communities.

Comparing our results with the findings of local studies, a similar study in Jeddah, SA, reported lower mean levels of TC and FBS, despite similar mean BMI in both studies. However, a higher rate of high TC levels was reported, with 32.5% of overweight and obese children having TC levels above 5.2 mmol/L, compared to 8% in our study. More interestingly, the mean TG in the study mentioned above was higher than our findings (1.8 versus 1), and the prevalence of high TG reached 49%, although investigators used a higher cutoff point in defining hypertriglyceridemia (2.2 mmol/L) [20].

Contrary to our findings, one local study found no relationship between obesity and changes in TG levels. The mean TC and LDL-C levels in our study correspond to the mean TC and LDL-C levels in morbidly obese children in that study [18]. Additionally, the study found no significant differences in metabolic profiles between genders and age groups, which is similar to another study [18]. However, a large local study that investigated 2,149 males and females aged 6-18 years reported significant differences in lipid profiles between males and females (P<0.0001) [26]. This difference may be attributable to the difference in sample size between the two studies.

The strengths of the study include the recent data and the variety of study areas, while its weaknesses include its retrospective design and the non-probability convenience sampling technique used to select the study sample.

## Conclusions

In conclusion, our study estimated the prevalence of abnormal lipid and FBS profiles in overweight and obese children and adolescents and found an increased risk of hypercholesterolemia, hypertriglyceridemia, and hyperglycemia, with no significant differences between age groups and genders. This study emphasizes the critical importance of early detection and intervention in childhood and adolescent obesity. As the prevalence of overweight and obese children is increasing worldwide, we recommend conducting more studies, especially in local settings, to detect and control dyslipidemia and hyperglycemia at earlier ages. This is the best strategy to avoid long-term consequences and protect children from the risk of cardiovascular morbidities and mortalities.

## References

[1] Sahoo K, Sahoo B, Choudhury AK, Sofi NY, Kumar R, Bhadoria AS (2015). Childhood obesity: causes and consequences. J Family Med Prim Care.

[2] Apovian CM (2016). Obesity: definition, comorbidities, causes, and burden. Am J Manag Care.

[3] Wang Y, Lobstein T (2006). Worldwide trends in childhood overweight and obesity. Int J Pediatr Obes.

[4] González-Álvarez MA, Lázaro-Alquézar A, Simón-Fernández MB (2020). Global trends in child obesity: are figures converging?. Int J Environ Res Public Health.

[5] Al Shehri A, Al Alwan I, Al Fattani A (2013). Obesity among Saudi children. Saudi j Obes.

[6] Al-Muhaimeed AA, Dandash K, Ismail MS, Saquib N (2015). Prevalence and correlates of overweight status among Saudi school children. Ann Saudi Med.

[7] Alturki HA, Brookes DS, Davies PS (2018). Comparative evidence of the consumption from fast-food restaurants between normal-weight and obese Saudi schoolchildren. Public Health Nutr.

[8] El Mouzan MI, Foster PJ, Al Herbish AS, Al Salloum AA, Al Omer AA, Qurachi MM, Kecojevic T (2010). Prevalence of overweight and obesity in Saudi children and adolescents. Ann Saudi Med.

[9] Abou Abbas O, AlBuhairan F (2017). Predictors of adolescents' mental health problems in Saudi Arabia: findings from the Jeeluna(®) national study. Child Adolesc Psychiatry Ment Health.

[10] Habbab RM, Bhutta ZA (2020). Prevalence and social determinants of overweight and obesity in adolescents in Saudi Arabia: a systematic review. Clin Obes.

[11] Al Dhaifallah A, Mwanri L, Aljoudi A (20151). Childhood obesity in Saudi Arabia: opportunities and challenges. Saudi J Obes.

[12] Al-Hussaini A, Bashir MS, Khormi M, AlTuraiki M, Alkhamis W, Alrajhi M, Halal T (2019). Overweight and obesity among Saudi children and adolescents: where do we stand today?. Saudi J Gastroenterol.

[13] Li L, Pérez A, Wu LT, Ranjit N, Brown HS, Kelder SH (2016). Cardiometabolic risk factors among severely obese children and adolescents in the United States, 1999-2012. Child Obes.

[14] Nasreddine L, Naja F, Tabet M (2012). Obesity is associated with insulin resistance and components of the metabolic syndrome in Lebanese adolescents. Ann Hum Biol.

[15] Mokha JS, Srinivasan SR, Dasmahapatra P, Fernandez C, Chen W, Xu J, Berenson GS (2010). Utility of waist-to-height ratio in assessing the status of central obesity and related cardiometabolic risk profile among normal weight and overweight/obese children: the Bogalusa Heart Study. BMC Pediatr.

[16] Nascimento H, Costa E, Rocha-Pereira P (2012). Cardiovascular risk factors in portuguese obese children and adolescents: impact of small reductions in body mass index imposed by lifestyle modifications. Open Biochem J.

[17] Kelishadi R, Mirmoghtadaee P, Najafi H, Keikha M (2015). Systematic review on the association of abdominal obesity in children and adolescents with cardio-metabolic risk factors. J Res Med Sci.

[18] Milyani AA, Al-Agha AE (2019). The effect of body mass index and gender on lipid profile in children and adolescents in Saudi Arabia. Ann Afr Med.

[19] Alaqeel AA (2019). Pediatric diabetes in Saudi Arabia: challenges and potential solutions. A review article. Int J Pediatr Adolesc Med.

[20] Alissa EM, Sutaih RH, Kamfar HZ, Alagha AE, Marzouki ZM (2023). Relationship between pediatric adiposity and cardiovascular risk factors in Saudi children and adolescents. Arch Pediatr.

[21] American Diabetes Association Professional Practice Committee (2022). 8. Obesity and Weight Management for the Prevention and Treatment of Type 2 Diabetes: Standards of Medical Care in Diabetes-2022. Diabetes Care.

[22] NCEP Expert Panel on Blood Cholesterol Levels in Children and Adolescents (1992). National Cholesterol Education Program (NCEP): highlights of the report of the expert panel on blood cholesterol levels in children and adolescents. Pediatrics.

[23] Rafii ZS, al Nasser AA, Budair A, Tufail M (1994). Establishing reference total serum cholesterol values for Saudi children. Ann Clin Biochem.

[24] Meral G, Uslu A, Yozgatli AÜ, Akçay F (2015). Association of body mass index and lipid profiles in children. Open J Pediatr.

[25] Korsten-Reck U, Kromeyer-Hauschild K, Korsten K, Baumstark MW, Dickhuth HH, Berg A (2008). Frequency of secondary dyslipidemia in obese children. Vasc Health Risk Manag.

[26] Tamimi W, Albanyan E, Altwaijri Y, Tamim H, Alhussein F (2012). Age- and gender-specific reference intervals for fasting blood glucose and lipid levels in school children measured with Abbott Architect c8000 chemistry analyzer. Indian J Clin Biochem.

